# Gasdermin E-mediated intestinal epithelial pyroptosis promotes chemically induced colitis in mice

**DOI:** 10.1093/gastro/goaf021

**Published:** 2025-03-18

**Authors:** Yi-Zhong Wu, Yao Xie, Lin Chen, Lei Ning, Xiao-Qi Hu, Xiao-Ping Xu

**Affiliations:** Department of Gastroenterology, Hunan Provincial People’s Hospital and The First Affiliated Hospital of Hunan Normal University, Changsha, Hunan, P. R. China; The Second Affiliated Hospital, Hengyang Medical School, University of South China, Hengyang, Hunan, P. R. China; The First People's Hospital of Xiangtan City, Xiangtan, Hunan, P. R. China; Department of Gastroenterology, Hunan Provincial People’s Hospital and The First Affiliated Hospital of Hunan Normal University, Changsha, Hunan, P. R. China; Department of Gastroenterology, Hunan Provincial People’s Hospital and The First Affiliated Hospital of Hunan Normal University, Changsha, Hunan, P. R. China; Department of Gastroenterology, Hunan Provincial People’s Hospital and The First Affiliated Hospital of Hunan Normal University, Changsha, Hunan, P. R. China

**Keywords:** ulcerative colitis, pyroptosis, gasdermin E, intestinal epithelial cell

## Abstract

**Background:**

Gasdermin E (GSDME) is a newly identified pyroptosis executioner and is upregulated in the intestinal epithelial cell (IEC) of ulcerative colitis (UC) patients. However, the effects of epithelial GSDME on UC remain unknown.

**Methods:**

Bone marrow chimera experiments were performed to investigate the role of GSDME in nonhematopoietic cells, mainly including IECs. An FITC-dextran assay was used to assess the integrity of the intestinal epithelial barrier.

**Results:**

*Gsdme^–/–^* chimeras that were reconstituted with wild-type bone marrow cells exhibited lower weight loss, disease activity index, colon shortening, and histology scores than wild-type chimeras after treatment with dextran sulfate sodium (DSS). However, *Gsdme*^+/+^ chimeras that were reconstituted with *Gsdme*-deficient bone marrow cells were not protected from DSS-induced colitis compared with wild-type chimeras. Importantly, DSS treatment activated Caspase-3 and cleaved GSDME to generate GSDME-N terminal fragments that are responsible for the induction of pyroptosis in IECs, but not in the intestinal lamina propria cell. Additionally, GSDME deficiency inhibited DSS-induced disruption of the intestinal epithelial barrier. Mechanistically, GSDME-mediated IEC pyroptosis is dependent on Caspase-3 activation, which is supported by the observation that the Caspase-3 inhibitor Z-DEVD-FMK inhibited DSS-induced GSDME cleavage in IECs.

**Conclusions:**

We show that GSDME-mediated epithelial pyroptosis contributes to the development of DSS-induced colitis by promoting intestinal inflammation and disrupting the intestinal epithelial barrier.

## Introduction

Ulcerative colitis (UC)—one clinical phenotype of inflammatory bowel disease (IBD)—is a chronic intestinal inflammatory disorder. In recent decades, the incidence of UC has greatly increased worldwide [1]. Its major symptoms include diarrhea, blood stool, and abdominal pain. Extensive inflammatory responses in the mucosa are the compelling feature in UC that leads it to be a kind of refractory intestinal disease [2]. However, the molecular mechanism of regulating abnormal mucosal inflammation in UC is not yet completely known.

Pyroptosis was initially identified simply as Caspase-1-mediated death, mainly in response to bacterial invasion [3]. Later findings that gasdermin D (GSDMD), as the pyroptotic substrate, is cleaved by activated Caspase-1/4/5/11 to generate the pyroptosis-inducing fragment GSDMD-N terminal (GSDMD-NT) to redefine pyroptosis as gasdermin-mediated programmed cell death [4, 5]. The gasdermin-NT in GSDMD binds membrane lipids and perforates the membrane, which causes cytoplasmic swelling and large bubbles that blow from the cell membrane with the rapid release of intracellular contents such as immunogenic damage-associated molecular patterns (DAMPs) and pro-inflammatory cytokines to induce strong inflammatory responses [6, 7].

Given the distinctive characteristic of pyroptosis in promoting inflammation [8], it is likely to play important roles in inflammatory diseases such as IBD. However, limited efforts have been directed toward the investigation of gasdermin-mediated pyroptosis in IBD. One report showed that GSDMD deficiency protects mice from dextran sulfate sodium (DSS)-induced colitis [9], whereas another report found that GSDMD exhibited anti-inflammatory effects in this chemically induced colitis model [10]. Gasdermins are mainly expressed in the epithelium of the gastrointestinal tract and skin [11, 12], which contain gasdermin A–E (GSDMA, GSDMB, GSDMC, GSDMD, GSDME) and DFNB59 in humans. The functional relevance and the effects of other pyroptosis executioners on colitis remain unknown.

GSDME, originally known as DFNA5 (Deafness, Autosomal Dominant 5) [12], is a newly identified pyroptosis executioner that is cleaved specifically by Caspase-3 activated by TNFα or chemotherapy drugs to generate the pyroptosis-inducing fragment GSDME-NT, thus switching Caspases-3-mediated non-inflammatory apoptosis to pro-inflammatory pyroptosis [13]. GSDME has been linked to the potential pathogenesis of some diseases such as deafness [14] and gastric and colonic cancers [15]. Previous studies have shown that loss of GSDME causes resistance to chemotherapy drugs in many cancer cells [16]. In our previous study, we showed that GSDME is upregulated in the intestinal epithelial cell (IEC) of UC patients [6]. However, the effects of intestinal epithelial GSDME on UC remain unknown. Here, we explored the functional relevance of epithelial GSDME in DSS-induced colitis in mice and investigated whether GSDME-mediated pyroptosis participates in the development of DSS-induced colitis.

## Materials and methods

### Antibodies and reagents

Anti-GSDME-NT (ab215191) and anti-NOD-like receptor thermal protein domain associated protein 3 (NLRP3; ab263899) antibodies were purchased from Abcam (Cambridge, UK). Anti-cleaved-Caspase-1 (89332), anti-cleaved-Caspase-3 (9661), and anti-cleaved-Caspase-8 (9429 and 9496) were purchased from Cell Signaling Technology (Boston, USA). Anti-zonula occludens-1 (ZO-1) antibody (21773–1-AP) was purchased from Proteintech (Chicago, USA). DSS (9011–19-8–1) was purchased from MP Biomedicals (California, USA). Recombinant murine tumor necrosis factor α (TNFα; 315-01A) were purchased from PeproTech (New Jersey, USA). Cycloheximide (C7698), a eukaryote protein synthesis inhibitor, was purchased from Sigma-Aldrich (Missouri, USA). Z-DEVD-FMK (HY-12466), a Caspase-3 inhibitor, was purchased from MedchemExpress (New Jersey, USA).

### Human samples

Endoscopic intestinal mucosal tissues were collected from UC patients and healthy controls at the Hospital Gastroenterology Unit of Hunan Provincial People’s Hospital. UC diagnoses were based on a standard combination of clinical, endoscopic, and histological assessment. The enrolled UC patients and healthy controls from January 2021 to December 2022 were excluded if they had cancer, hypertension, diabetes, lung disease, and cardiovascular disease. The severity in UC was graded from 0 to 5 according to Geboes criteria [17]. All tissues were collected from consenting individuals according to the protocols approved by the Ethics Committee of Hunan Provincial People’s Hospital. Demographic characteristics are shown in Supplementary Tables 1 and 2.

### Mouse models

Wild-type (WT) C57BL/6 and *Gsdme*^–/–^ mice (C57BL/6 strain) were bred and maintained in a specific-pathogen-free facility. All animal study protocols were approved by the Institutional Animal Care and Use Committee at Hunan Provincial People’s Hospital (NO. 2020180). The *Gsdme*^–/–^ mice were kindly gifted by Professor Gao Tan (Nanfang Hospital, Guangzhou, China). The WT and *Gsdme*^–/–^ mice were littermates and co-housed throughout the experiments. DSS-induced colitis models were constructed by using the method adapted from a published procedure [18]. Briefly, 8- to 10-week-old male *Gsdme*^–/–^ mice and WT littermates were given 2.5% DSS dissolved in drinking water for 7 days to induce colitis. To inhibit the activation of Caspase-3, a specific inhibitor Z-DEVD-FMK in phosphate buffered saline (PBS) containing 1% DMSO was given through intraperitoneal injection at a concentration of 7 mg/kg per day. Colon tissues were embedded in paraffin and stained with hematoxylin-eosin. Histology scores were carried out [18] and were assessed by two pathologists individually.

### Bone marrow transplant model

A bone marrow transplant model was generated as described previously [19]. Briefly, bone marrow cells that were collected from the tibias and femurs of donor *Gsdme^+/+^* or *Gsdme*^–/–^ mice were suspended in PBS at a final concentration of 3.0 × 10^4^ cells/μL. Recipient (*Gsdme*^+/+^ or *Gsdme*^–/–^) mice were irradiated with 10 Gy, intravenously injected with 100 μL of cell suspension, and then administered water containing 2 g/L neomycin sulfate ad libitum for 2 weeks. Six weeks after transplantation, the mice were used in the experiments.

### Isolation of intestinal epithelial cells

Biopsy samples were processed immediately and IECs were purified by using enzyme digestion as described previously [20]. IECs from *Gsdme*^–/–^ mice and WT littermates 7 days after DSS treatment were isolated by using isolation buffer (30 mM EDTA and 1 mM dithiothreitol (1 mM DTT)). Briefly, the intestinal tract was dissected and the fecal contents were removed by repeated cleaning with cold PBS without calcium and magnesium. The intestinal tract was cut into small pieces, 0.5 mg/mL of isolation buffer was added, and they were cultured in a water bath at 37°C for 30 min. After centrifugation, the supernatant was collected and precipitated for further digestion with 0.5 mg/mL of isolation buffer for 40 min. After digestion for a total of 70 min, the supernatant was combined with the precipitation and filtered by using a 70-μM cell filter (BD Falcon 352350). After centrifugation at 500 ×*g* for 5 min, the precipitation was digested with 1 mL of trypsin for 15 min and the supernatant was filtered by using a 35-μM cell filter (BD Falcon 352235). After centrifugation at 500 ×*g* for 5 min, the precipitated cells were suspended with PBS plus 0.01% BSA to obtain intestinal epithelial cells.

### Isolation of lamina propria cells

Enteric lamina propria cells were separated from the colons of *Gsdme*^–/–^ mice and WT littermates 7 days after DSS treatment. The colon tissues were cut into small pieces and then incubated with RPMI medium supplemented with fetal bovine serum (FBS) and 5 mM of EDTA at 37°C for 60 min with shaking. After removal of the epithelial layer, the remaining colon segments were incubated at 37°C with RPMI medium containing 500 μg/mL of collagenase type IV (Sigma) for 90 min. The supernatant was passed through a 70-μM cell strainer to isolate the lamina propria cells.

### RNA sequencing

RNA sequencing was used for measurement of the mRNA expression levels of many cytokines in the colons of the *Gsdme*^–/–^ mice and WT littermates 7 days after DSS treatment. The total RNA isolated was analysed by using BGI RNA sequencing. The original transcriptome reading was mapped to the reference genome (GRCm38/mm10) by using Bowtie. The gene expression level was quantitatively analysed by using the RSEM software package. Significantly affected genes were obtained by setting a fold change of >2 and error detection rate threshold of 0.05. The results were summarized as a heat map.

### Real-time polymerase chain reaction

Quantitative real-time polymerase chain reaction (PCR) was performed as previously described [21]. The mRNA level of the target genes was normalized to that of glyceraldehyde-3-phosphate dehydrogenase (GAPDH). The primers that were used for the target genes mentioned in the article are shown in Supplementary Table 3.

### Western blotting

Western blotting was performed as previously described [21]. GAPDH was used as an endogenous control. Anti-GSDME-NT and anti-NLRP3 antibodies were diluted at a ratio of 1:2,000. Anti-cleaved Caspase1, anti-cleaved Caspase3, anti-cleaved Caspase8, and anti-cleaved GSDMD were diluted at a ratio of 1:1,000. The appropriate secondary antibodies were diluted at a ratio of 1:3,000. ImageJ software was used to quantify and analyse the density of the protein bands.

### Fluorescein isothiocyanate-dextran assay

*Gsdme* ^–/–^ mice and WT littermates were fed with 7-day DSS to induce colitis. Subsequently, they were given fluorescein isothiocyanate (FITC)-conjugated dextran (4,000 MW) by using gavage administration (at 0.2 mg/g of body weight). After 4 h, the concentration of FITC-dextran in blood serum was measured.

### Statistical analysis

**Figure 1.**
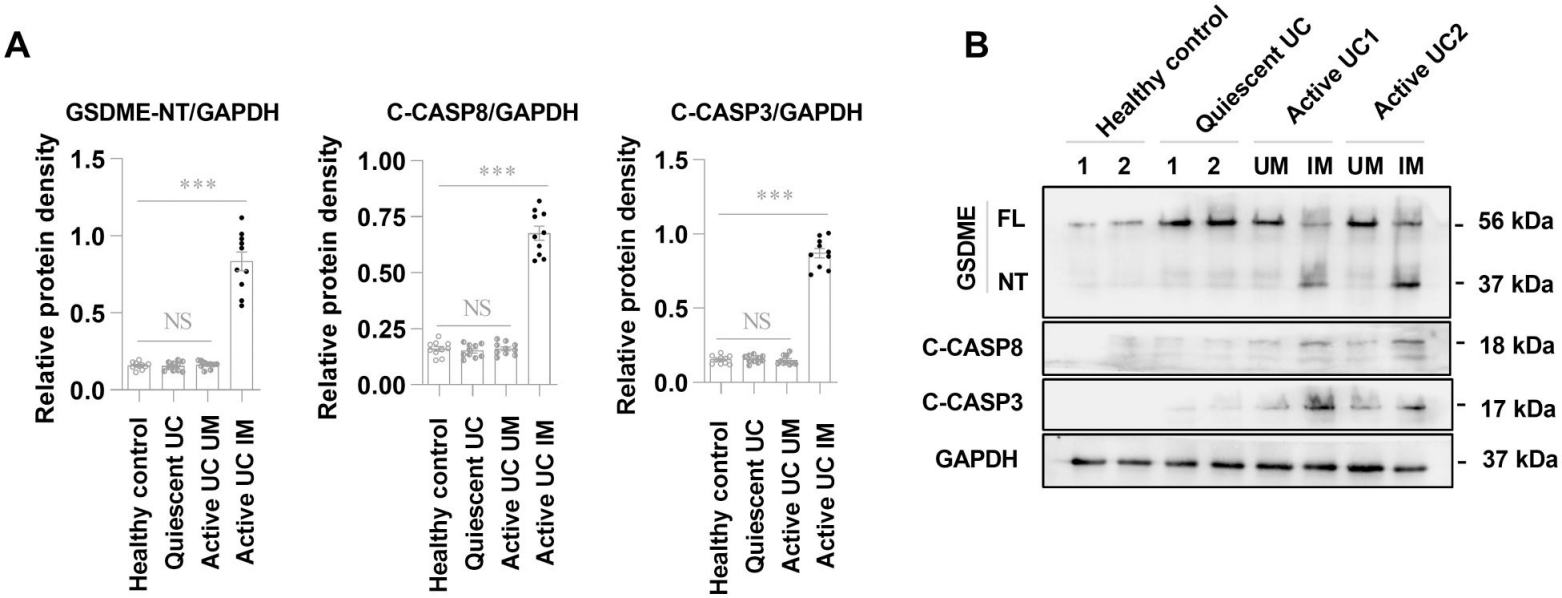
GSDME is cleaved in the inflamed mucosa of UC. (A) and (B) Western blot examination of the levels of GSDME cleavage and Caspase-8 and Caspase-3 activation in the intestinal mucosa from healthy controls (*n* = 10), quiescent UC patients (*n* = 10), and active UC patients (*n* =10). Full-length GSDME (GSDME-FL) has a molecular weight of 56 kDa and GSDME-NT is 37 kDa. (A) Quantitative analyses of proteins. (B) Representative images. ***P < 0.001. UM = uninflamed mucosa, IM = inflamed mucosa, C-CASP3 = Cleaved-Caspase-3, C-CASP8 = Cleaved-Caspase-8, GSDME = gasdermin E, UC = ulcerative colitis, NS = not significant.

Unless indicated otherwise, statistical analyses were accomplished by using GraphPad Prism software. Data in Figure 1A are presented as mean ± SEM and statistical significance was determined by using a one-way Analysis of Variance (ANOVA) test. All animal experimental data are presented as mean ± SEM and statistical significance was determined by using a two-tailed Student’s *t*-test. *P*-values of <0.05 were considered significant.

## Results

### GSDME-mediated pyroptosis positively associates with abnormal mucosal inflammation in UC

To investigate the involvement of GSDME-mediated pyroptosis in mucosal inflammation in UC, the pyroptosis-inducing fragment GSDME-NT was measured in the mucosa of active and quiescent UC patients and healthy controls. GSDME-NT was markedly detected in the inflamed colonic mucosa of active UC patients but not in the uninflamed mucosa of active or quiescent UC patients or controls (Figure 1A and B), indicating that GSDME-mediated pyroptosis is involved in abnormal mucosal inflammation in UC. In addition, Caspase-8 and Caspase-3 were activated in the inflamed mucosa of active UC patients (Figure 1A and B). These results indicate that GSDME-mediated pyroptosis contributes to the pathogenesis of UC.

### GSDME-mediated pyroptosis participates in DSS-induced colitis

**Figure 2.**
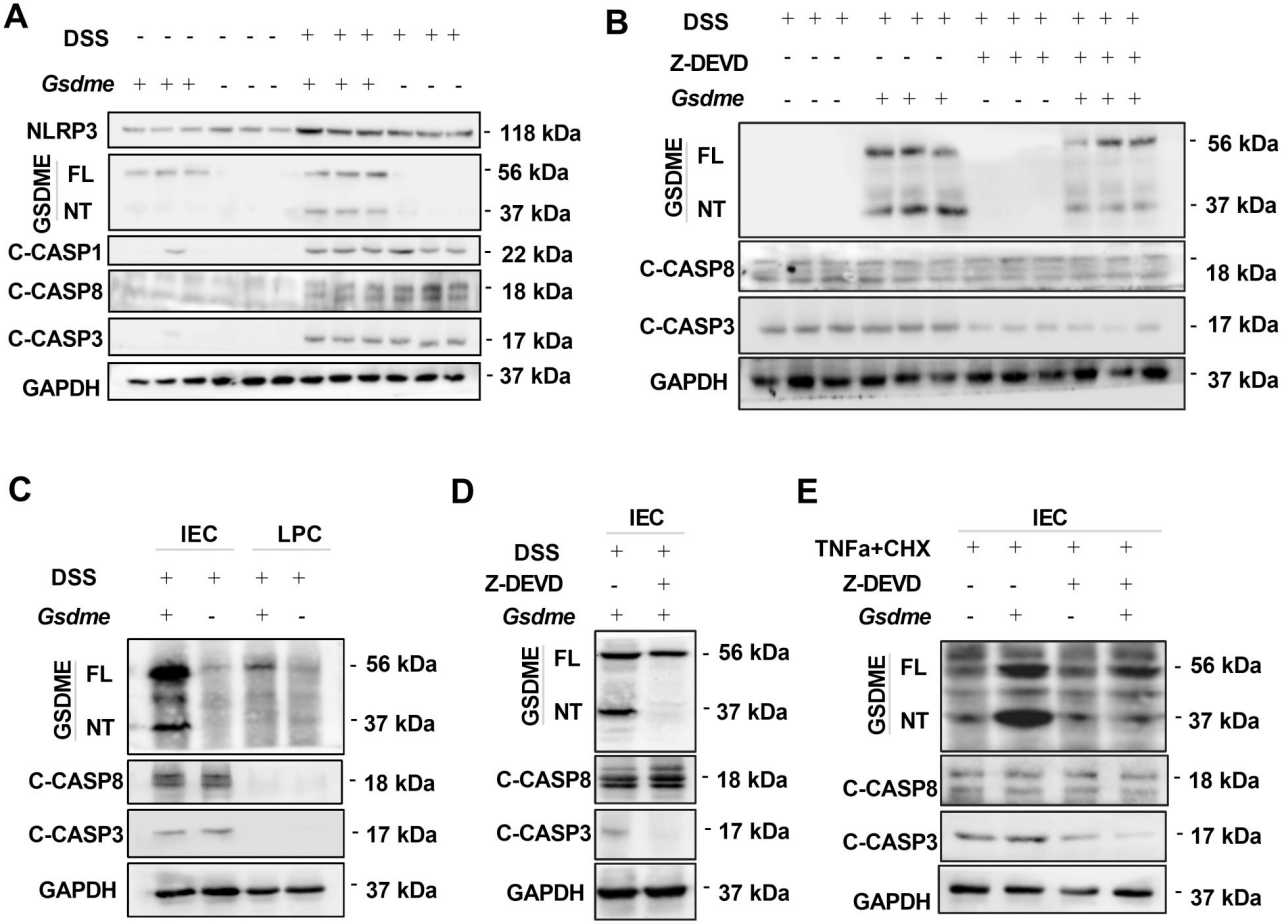
GSDME mediates IEC pyroptosis through the Caspase3-dependent pathway. (A) and (B) Representative immunoblot images of GSDME cleavage, Caspase-1/3/8, and NLRP3 expression in the intestinal mucosa from *Gsdme*^–/–^ mice and wild-type (*Gsdme*^+/+^) control littermates that were challenged with DSS with or without Z-DEVD-FMK. Full-length GSDME (GSDME-FL) has a molecular weight of 56 kDa and GSDME-NT is 37 kDa. (C) and (D) Representative immunoblot images of GSDME cleavage and Caspase-3 and Caspase-8 activation in the IEC and LPC from *Gsdme*^–/–^ mice and wild-type littermates that were challenged with DSS with or without Z-DEVD-FMK. (E) Representative immunoblot images of GSDME cleavage and Caspase-3 and Caspase-8 activation in isolated IECs from *Gsdme*^–/–^ mice and wild-type littermates that were treated with TNFα (50 ng/mL) plus CHX (20 μg/mL) for 8 h to induce pyroptosis *in vitro*. GSDME-FL has a molecular weight of 56 kDa and GSDME-NT is 37 kDa. All data shown are representative of three independent experiments. C-CASP3 = Cleaved-Caspase-3, C-CASP8 = Cleaved-Caspase-8, IEC = Intestinal epithelial cell, LPC = lamina propria cell, Z-DEVD = Z-DEVD-FMK, CHX = cycloheximide, GSDME = gasdermin E.

GSDME can switch Caspases-3-mediated non-inflammatory apoptosis to pro-inflammatory pyroptosis [13]. In our previous study, we found that GSDME deficiency protected mice from DSS-induced colitis [6]. Therefore, we speculated that GSDME may promote DSS-induced colitis through mediating pyroptosis. As expected, DSS treatment activated Caspase-3 and Caspase-8, and cleaved GSDME, and the pyroptosis-inducing fragment GSDME-NT was markedly detected in the colons of WT mice (Figure 2A). However, activated Caspase-3 and GSDME-NT were decreased in Z-DEVD-FMK-treated mice (Figure 2B). Although DSS treatment activated NLRP3 and Caspase-1/3/8, there were no significant differences between GSDME-KO mice and WT littermates (Figure 2A). Collectively, these data indicate that GSDME-mediated and Caspase-3-dependent pyroptosis participates in DSS-induced colonic inflammation.

### GSDME deficiency restricts DSS-induced colonic inflammatory responses

**Figure 3.**
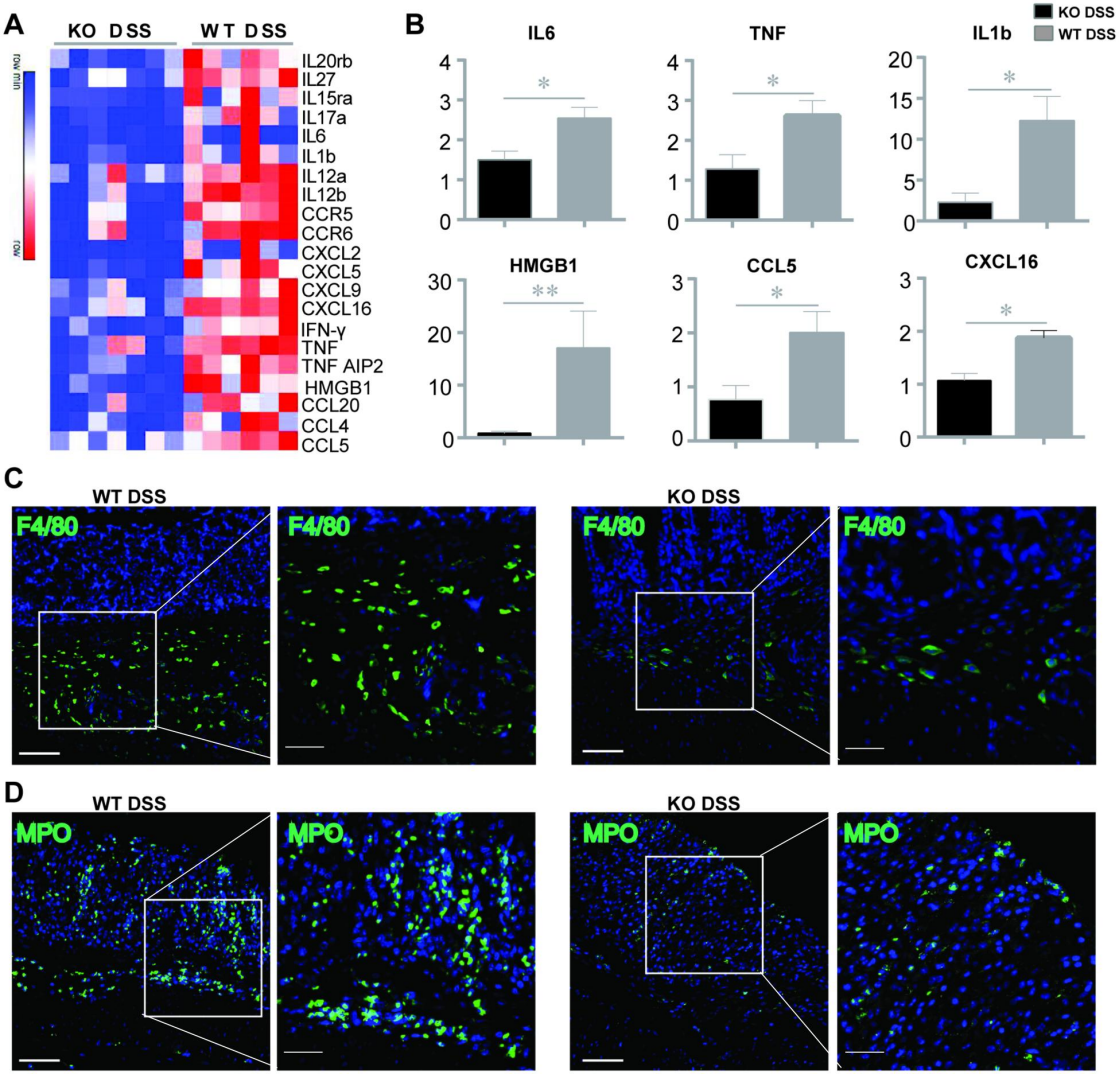
GSDME deficiency restricts DSS-induced inflammatory responses in colonic tissues. *Gsdme*^–/–^ (KO) mice and WT control littermates were given 2.5% DSS in drinking water for 7 days to induce acute experimental colitis (*n* = 6 per group). (A) RNA sequencing and (B) real-time PCR analyses of indicated genes in whole colonic homogenates. (C) and (D) Representative immunofluorescence images of MPO and F4/80 immunostaining in colon tissues (scale bars: 200 μm). White boxes represent enlarged images (scale bars: 50μm). Data are shown as mean ± SEM from six mice in each group. Data shown are representative of three independent experiments. *P < 0.05; **P < 0.01. GSDME = gasdermin E, KO = knockout, DSS = dextran sulfate sodium, WT = wild-type, MPO = myeloperoxidase, PCR = polymerase chain reaction.

IBD is an inflammation-driven intestinal disease with high levels of inflammatory cell infiltration into local mucosal tissues [22]. We found that DSS treatment dramatically induced the colonic mucosal pro-inflammatory cytokine, DAMP, and chemokine expression in WT mice, such as TNFα, HMGB1, IL1b, IL6, CCL5, and CXCL16, but the induction of these pro-inflammatory mediators was significantly attenuated in GSDME-KO mice (Figure 3A and B), indicating that the GSDME-KO colons are less inflamed than the WT colons. Furthermore, the colons of GSDME-deficient mice contained fewer infiltrating MPO^+^ neutrophils and F4/80^+^ macrophages in DSS-induced colitis (Figure 3C and D), indicating that GSDME contributes to DSS-induced colitis through recruiting inflammatory immune cells and initiating inflammatory responses.

### Absence of GSDME in nonhematopoietic cells relieves DSS-induced colitis

**Figure 4.**
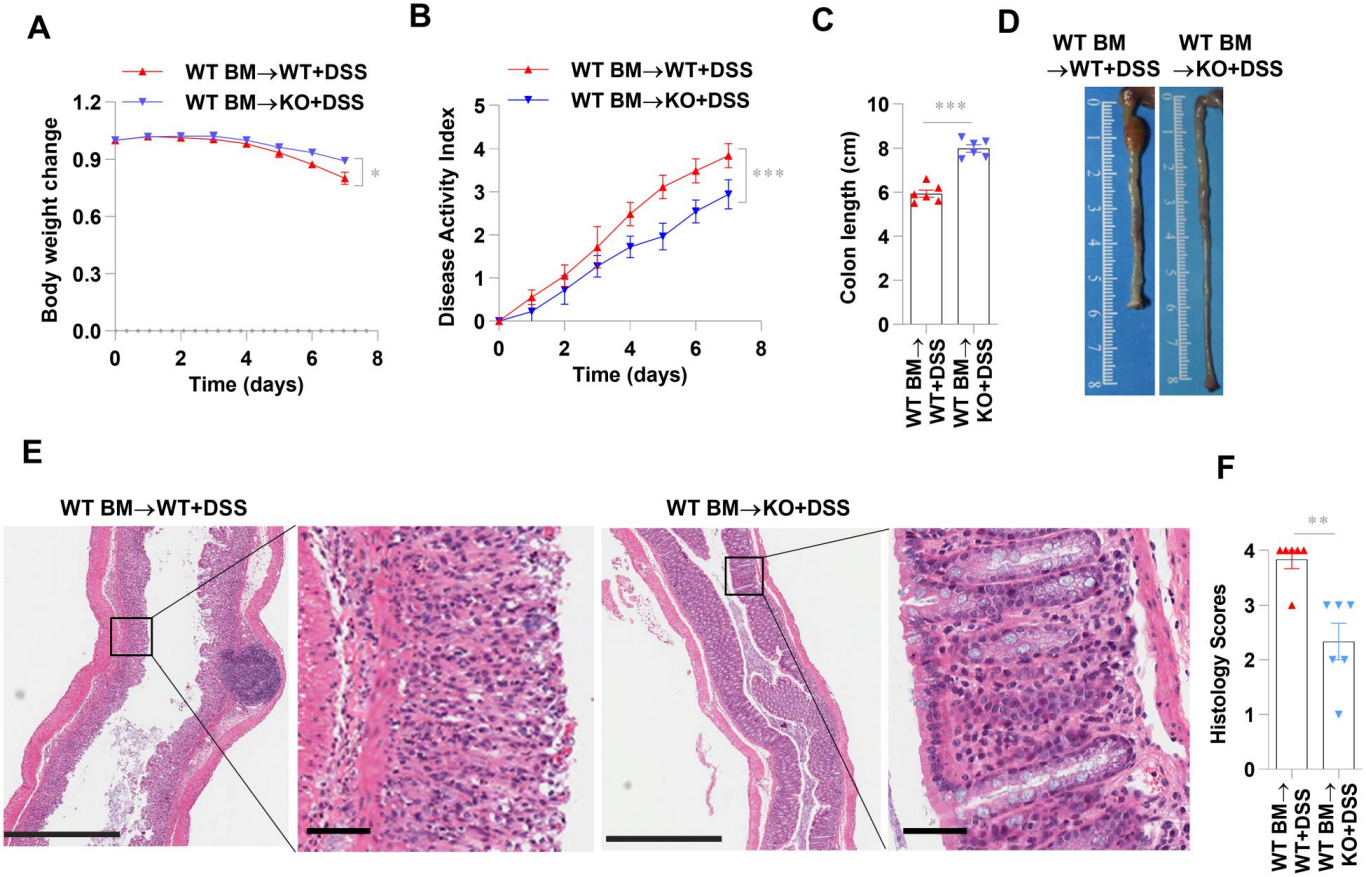
Absence of GSDME in nonhematopoietic cells relieves DSS-induced colitis. Non-lethally irradiated *Gsdme*^–/–^ (KO) mice and WT littermates received WT BM cells to generate chimeric mice. Then these chimeric mice were given 2.5% DSS in drinking water for 7 days to induce acute experimental colitis (*n* = 6 per group). (A) and (B) Body weight changes and disease activity index were measured daily. (C) and (D) Colon length values of the mice were measured after 7 days of DSS treatment. (E) and (F) Representative HE-stained sections of middle colon tissues collected after 7 days of DSS treatment (scale bars: 1 mm). Black boxes represent enlarged images (scale bars: 200 μm). Histopathology changes reflected by semi-quantitative scores. *P < 0.05; **P < 0.01; ***P < 0.001. All data shown are representative of three independent experiments. GSDME = gasdermin E, KO = knockout, DSS = dextran sulfate sodium, WT = wild-type, BM = bone marrow, HE = hematoxylin-eosin.

To investigate the relevant cell compartment that is responsible for the diminished intestinal inflammation in the GSDME-KO mice, bone marrow chimera experiments were performed. Non-lethally irradiated GSDME-KO mice and littermate controls were reconstituted with bone marrow cells from WT mice. The reconstituted mice with GSDME deficiency in nonhematopoietic cells exhibited lower weight loss, disease activity index, colon shortening, and histology scores than the WT chimeras after DSS treatment (Figure 4A–F). However, *Gsdme*^+/+^ mice that received *Gsdme*^–/–^ bone marrow cells were not protected from DSS-induced colitis as compared with *Gsdme*^+/+^ mice that received *Gsdme*^+/+^ bone marrow cells (Supplementary Figure 1). Collectively, these data implicate that the lack of GSDME in the epithelial or stromal cells protected the mice against DSS-induced colitis.

Previous studies have shown that gasdermins are mainly expressed in the epithelium of the gastrointestinal tract and skin [12]. A previous study showed that GSDME is mainly expressed in the IEC, but to a lesser extent in the intestinal lamina propria cell [8]. However, DSS treatment activated Caspase-8 and Caspase-3, and cleaved GSDME in IECs, but not in lamina propria cells (Figure 2C). Taken together, these results suggest that IECs may have been primarily responsible for the reduced intestinal inflammation in the GSDME-KO mice.

### GSDME deficiency reduces DSS-induced intestinal barrier disruption

**Figure 5.**
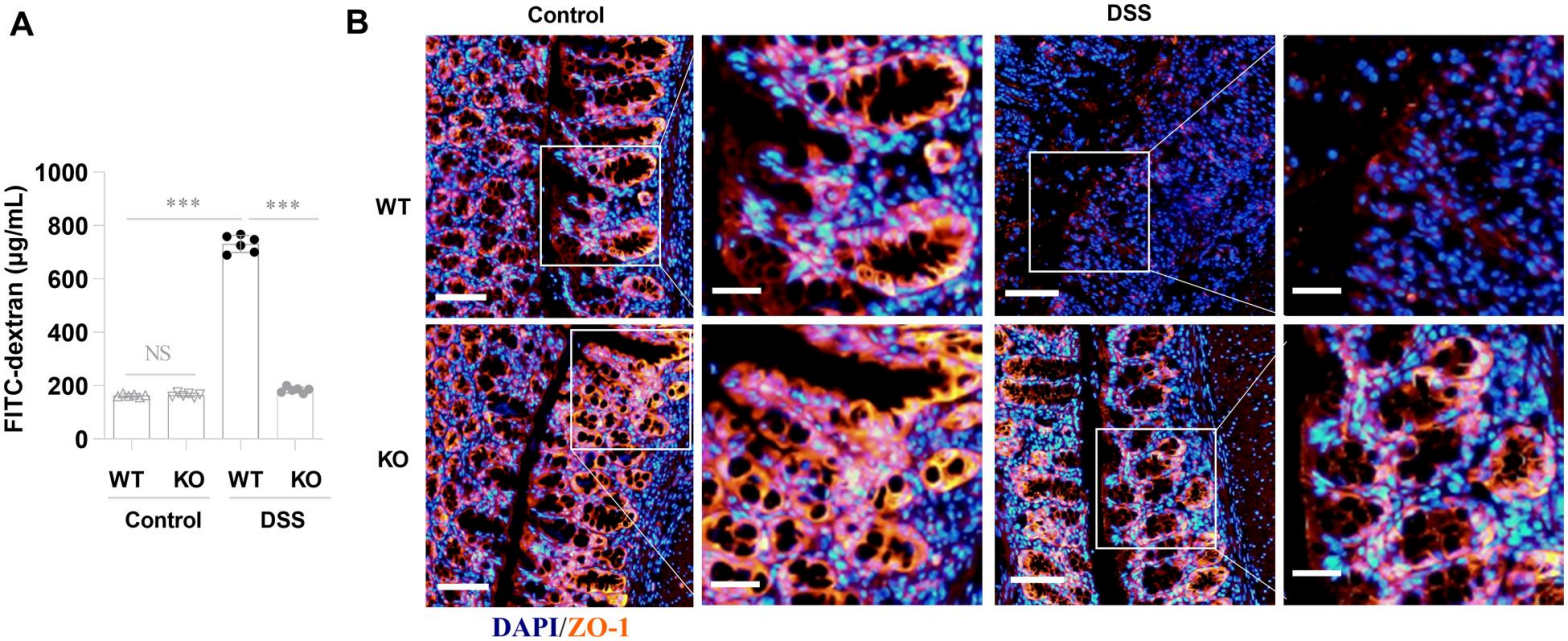
GSDME deficiency reduces DSS-induced intestinal barrier disruption. (A) *Gsdme*^–/–^ (KO) mice and WT control littermates were given drinking water (control) or 2.5% DSS in drinking water, then these mice were fed with FITC-dextran (0.2 mg/g), 4 h before sacrifice on day 7 and the serum levels of FITC-dextran were detected by using enzyme linked immunosorbent assay (ELISA). Data are shown as mean ± SEM from six mice in each group. (B) Representative immunofluorescence images of cell tight junction protein ZO-1 immunostaining in colon tissues from the mice (scale bars: 100 μm). White boxes represent enlarged images (scale bars: 20μm). Data shown are representative of three independent experiments. ***P < 0.001. GSDME = gasdermin E, KO = knockout, DSS = dextran sulfate sodium, WT = wild-type, FITC = fluorescein isothiocyanate, ZO-1 = zonula occludens-1, NS = not significant.

It has been reported that intestinal barrier disruption is involved in IBD in patients and experimental colitis in mice [23]. To investigate whether GSDME affects intestinal barrier disruption in the DSS-induced colitis model, we determined FITC-dextran in the serum of GSDME-KO mice and WT control littermates 7 days after DSS treatment. The KO and WT mice exhibited similar epithelial permeability before DSS treatment (Figure 5A). However, the GSDME-KO mice displayed lower serum FITC-dextran concentrations than the WT mice after DSS treatment (Figure 5A), indicating that GSDME is important in the control of intestinal epithelial permeability upon DSS treatment. The tight junction protein ZO-1 that is vital for maintaining intestinal epithelial integrity [24, 25] has been used to indicate the intestinal epithelial integrity in the experimental colitis. After the DSS treatment, the colons of the WT mice had decreases in ZO-1, but the colons of the GSDME-KO mice displayed relatively normal ZO-1 levels in the epithelium (Figure 5B). Collectively, these data indicate that GSDME promotes DSS-induced intestinal epithelial barrier disruption.

### GSDME mediates IEC pyroptosis through a caspase-3-dependent pathway

A recent study has demonstrated that GSDME is specially cleaved by activated Caspase-3 in response to TNFα plus cycloheximide (CHX) to generate GSDME-NT that is responsible for inducing pyroptosis in cancer cells [13]. To investigate whether GSDME-mediated pyroptosis is Caspase-3-dependent in DSS-challenged IECs, we determined GSDME-NT, cleaved-Caspase-8, and cleaved-Caspase-3 in IECs that were isolated from WT mice after DSS treatment with or without Z-DEVD-FMK. GSDME cleavage, Caspase-3 activation, but not Caspase-8 activation were inhibited by Z-DEVD-FMK in DSS-challenged IECs (Figure 2D), indicating that DSS-induced and GSDME-mediated pyroptosis is dependent on Caspase-3. Similarly, after TNFα plus CHX treatment *in vitro*, GSDME-NT, cleaved-Caspase-3, and cleaved-Caspase-8 could be detected in IECs that were isolated from WT, but Z-DEVD-FMK inhibited the cleavage of GSDME and Caspase-3 activation (Figure 2E). Collectively, these data indicate that GSDME-mediated IEC pyroptosis is Caspase-3-dependent.

## Discussion

In this study, we showed that GSDME-mediated intestinal epithelial pyroptosis contributes to UC in humans and mice. We found that the pyroptosis-inducing fragment GSDME-NT was obviously detected in the inflamed mucosa, but not in the uninflamed mucosa. Moreover, GSDME deficiency in mice restricted DSS-induced IEC pyroptosis and maintained the integrity of the intestinal epithelial barrier, leading to experimental colitis resistance.

A previous study found that GSDME was mainly expressed in IECs [8]. In this study, we found that GSDME deficiency in the nonhematopoietic cells significantly alleviated DSS-induced colitis. In addition, DSS treatment markedly induced Caspase-3 activation and GSDME cleavage in IECs, which were inhibited by the Caspase-3 inhibitor Z-DEVD-FMK. Recent reports demonstrated that GSDME is specially cleaved by activated Caspase-3 to generate GSDME-NT that is responsible for inducing pyroptosis [13]. These findings show that GSDME-mediated and Caspase-3-dependent pyroptosis in IECs is responsible for DSS-induced colitis.

GSDME-mediated pyroptosis is believed to induce subsequent inflammatory responses by releasing cellular contents, such as immunogenic DAMPs [5, 14]. In our previous study, we found that HMGB1 expression and release from IECs were significantly increased in DSS-treated WT mice compared with GSDME-KO mice [6].

Once extracellular, HMGB1 can trigger inflammatory responses [26]. Collectively, our findings show that the DAMP molecule HMGB1 that is released from pyroptotic IECs promotes intestinal inflammation.

IECs form a barrier that segregates the intestinal bacteria from the mucosal immune system. Current evidence suggests that IEC barrier disruption contributes to exaggerated mucosal immune responses to the gut bacteria in IBD [27]. Our FITC-dextran permeability experiment indicates that GSDME deficiency is helpful for maintaining the integrity of the IEC barrier, which resulted in the resistance of GSDME-KO mice to experimental colitis. In addition, we also found that GSDME significantly increased immune cell infiltration into the intestinal mucosa.

The main limitation of this study is that the pyroptosis of intestinal epithelial cells was not directly measured. While Caspase-3 has been implicated in pyroptosis, this caspase plays a role in other forms of cell death, such as apoptosis. Further, activation of gasdermins does not necessarily lead to pyroptosis. In some cases, gasdermins induced cellular membrane permeability through pore formation, but this pore formation is reversible. Thus, although GSDME cleavage and Caspase-3 activation were detected in the inflamed mucosa of UC and DSS mice, these imply that pyroptosis has occurred, but other forms of death cannot be ruled out.

## Conclusions

In summary, we show that GSDME-mediated IEC pyroptosis promotes intestinal inflammation and disrupts the mucosal epithelial barrier. In view of the positive relationship between GSDME cleavage and colitis severity in UC patients, GSDME-NT may be a predictive marker of active UC. It will be of interest to investigate whether pyroptosis-activating genetic mutations that affect GSDME cleavage contribute to susceptibility to UC. Targeted inhibition of GSDME-mediated pyroptosis with agents may offer a useful therapeutic approach for UC treatment.

## Supplementary Material

goaf021_Supplementary_Data
