# Supplementary material for: Gasdermin E-mediated intestinal epithelial pyroptosis promotes chemically induced colitis in mice
Source: Gastroenterol Rep (Oxf). 2025 Mar 18;13:goaf021. doi: 10.1093/gastro/goaf021 (PMC11919448; doi:10.1093/gastro/goaf021)
Supplement: goaf021_Supplementary_Data [file goaf021_supplementary_data.docx]

**Gasdermin E-mediated intestinal epithelial pyroptosis promotes chemically induced ulcerative colitis in mice**

**Supplementary figures and tables**


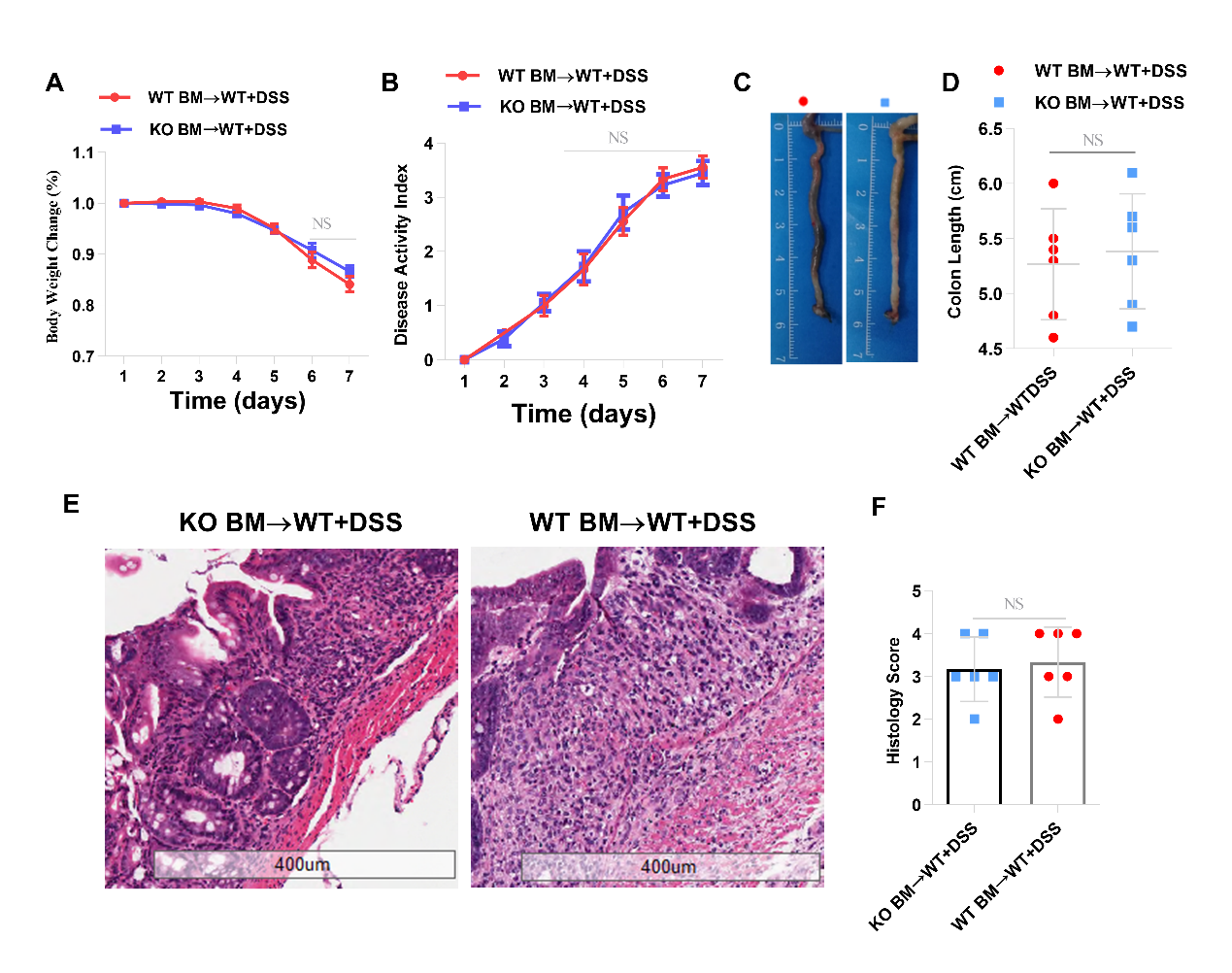


**Supplementary Figure 1 The severity of DSS-induced colitis is not dependent on GSDME in bone marrow-derived immune cells**

Gsdme^+/+^ mice receiving Gsdme^-/-^ bone marrow cells (KO BM→WT ) and Gsdme^+/+^ mice receiving Gsdme^+/+^ bone marrow cells (WT BM→WT) were fed with DSS for 7 days to induce colitis (6 per group). (**A**) Body Weight Change, (**B**) Disease Activity Index, (**C and D**) colon length and (**E and F**) histology scores were measured. Data are representative of three independent experiments.

KO: knockout, DSS: dextran sulfate sodium, WT: wild-type, BM: bone marrow, NS: not significant

**Supplementary Table 1 Demographic characteristics of the study population**

|  | **UC (60)** | **Controls (40)** |
| --- | --- | --- |
| **Male, n (%)** | 34 (56.67%) | 25 (62.5%) |
| **Age (years)** | 46.12 ± 15.75 (14–80) | 33.65 ± 10.89 (17–62) |
| **Disease duration (months)** | 39.7 ± 51.02 (0–240) | _ |
| **Age at diagnosis (years)**  **Treatment**  **TNF antagonist use**  **Aminosalicylates**  **Immune modulator use**  **Corticosteroids**  **None**  **smoking**  **Pathological severity**  **Grade 0–1**  **Grade 2–5** | 43.38 ± 16.03 (13–80)  10  40  8  22  6  35  18  42 | _  _  _  _  _  _  _  28  _  _ |

**Supplementary Table 2 Clinical characteristics of the study population**

|  | **UC** | **Controls** |
| --- | --- | --- |
| **Disease extent, n (%)**  Proctosigmoiditis  Left-sided colitis  Pancolitis | 14 (23.3%)  14 (23.3%)  32 (53.3%) | - |
| IBD-related surgery history | 13 (21.7%) | - |

**Supplementary Table 3 Primer sequences for real-time PCR**

| **Gene** | **Fw_sequence** | **Rv_sequence** |
| --- | --- | --- |
| **HMGB1** | GGCGAGCATCCTGGCTTATC | GGCTGCTTGTCATCTGCTG |
| **CCL5** | GCTGCTTTGCCTACCTCTCC | TCGAGTGACAAACACGACTGC |
| **CXCL16** | CCTTGTCTCTTGCGTTCTTCC | TCCAAAGTACCCTGCGGTATC |
| **IL1b** | GCAACTGTTCCTGAACTCAACT | ATCTTTTGGGGTCCGTCAACT |
| **IL6** | GTGGCTAAGGACCAAGACCA | ATAACGCACTAGGTTTGCCGA |
| **TNFα** | CCCTCACACTCAGATCATCTTCT | GCTACGACGTGGGCTACAG |
| **GAPDH** | TGTGTCCGTCGTGGATCTGA | TTGCTGTTGAAGTCGCAGGAG |
